# Comparison of Histological Sample Volumes among Various Endoscopic Ultrasound-Guided Biopsy Needles

**DOI:** 10.3390/jcm10163560

**Published:** 2021-08-13

**Authors:** Kosuke Takahashi, Ichiro Yasuda, Tatsuyuki Hanaoka, Yuka Hayashi, Iori Motoo, Shinya Kajiura, Takayuki Ando, Haruka Fujinami, Kazuto Tajiri, Johji Imura, Eisuke Ozawa, Satoshi Miuma, Hisamitsu Miyaaki, Kazuhiko Nakao

**Affiliations:** 1 Third Department of Internal Medicine, University of Toyama, Toyama 930-0194, Japan; takapochi0809@gmail.com (K.T.); T.Hanaoka.com@outlook.jp (T.H.); yukaberry0822@yahoo.co.jp (Y.H.); iori4869@med.u-toyama.ac.jp (I.M.); d12433@med.u-toyama.ac.jp (S.K.); taando33@gmail.com (T.A.); haruka52@med.u-toyama.ac.jp (H.F.); tajikazu@med.u-toyama.ac.jp (K.T.); 2 Department of Gastroenterology and Hepatology, Graduate School of Biomedical Sciences, Nagasaki University, Nagasaki 852-8501, Japan; eisukeozawa@nifty.com (E.O.); miuma1002@gmail.com (S.M.); hmiya0629@yahoo.co.jp (H.M.); kazuhiko@nagasaki-u.ac.jp (K.N.); 3 Department of Diagnostic Pathology, Faculty of Medicine, Academic Assembly, University of Toyama, Toyama 930-0194, Japan; imura@med.u-toyama.ac.jp

**Keywords:** pancreatic cancer, endoscopic ultrasound-guided fine-needle biopsy, Franseen needle, 19-gauge, 22-gauge

## Abstract

Endoscopic ultrasound-guided fine-needle biopsy (EUS-FNB) enables easy and accurate pathological assessment. Here, we compared and assessed the area of samples on glass slides for three needle types: a 19-gauge Franseen needle (Acquire, Boston Scientific, Natick, MA, USA), a 22-gauge Franseen needle, and a 19-gauge fine-needle aspiration (FNA) needle (EZ Shot 3 Plus; Olympus, Tokyo, Japan). Among patients with suspected pancreatic cancer, with a ≥20 mm tumor located in the pancreatic body and tail, and who underwent EUS-FNA or FNB between June 2018 and March 2020, 10 were randomly selected to test each needle. The areas of histological tissue and blood clot samples were measured using the BZ-X800 imaging software (Keyence Corporation, Osaka, Japan). Baseline patient characteristics and pathological sample data showed no significant differences among the needles. The 19-gauge Franseen needle obtained significantly more histological tissue samples than the 19-gauge conventional needle (*p* = 0.010) and 22-gauge Franseen needle (*p* = 0.008). Conversely, there was no significant difference between the 19-gauge conventional needle and 22-gauge Franseen needle (*p* = 0.838) in this regard. The 19-gauge Franseen needle could collect more samples than the other needles, contributing to giving a more precise pathological diagnosis and more information, including genomic profiling.

## 1. Introduction

Endoscopic ultrasound-guided fine-needle aspiration (EUS-FNA) is a safe and efficient procedure for pancreatic masses [1,2]. In a meta-analysis that focused on the EUS-FNA of pancreatic masses, the sensitivity for diagnosing the correct etiology was 86.8% (95% CI, 85.5–87.9%) and the specificity was 95.8% (95% CI, 94.6–96.7%) [3]. Furthermore, endoscopic ultrasound-guided fine-needle biopsy (EUS-FNB) has recently gained popularity, as it enables physicians to obtain more pathological samples and easily establish a more accurate pathological assessment [4,5]. In Japan, a needle with three novel symmetrical heels, called a Franseen needle, has become available for EUS-FNB. Currently, the strategy for the management of pancreatic cancer is rapidly changing. Precision medicine using genetic profiles, including microsatellite instability (MSI), has been actively carried out for unresectable advanced pancreatic cancer [1]. By contrast, it has been reported that the diagnostic accuracy of EUS-FNA for pancreatic cancer is lower than that for other pathologies as a result of the intense stromal desmoplastic reaction [6,7]. Therefore, enough samples should be obtained to enable a histological diagnosis of pancreatic cancer. Several studies have already reported the benefits of EUS-FNB for histological assessment [8,9,10]; however, there are no studies focusing on pancreatic cancer. In addition, no studies have compared the actual sample volume between various EUS-FNA and EUS-FNB needles using dedicated measuring equipment. Therefore, we compared and assessed the area of samples on glass slides from three types of needles: 19-gauge and 22-gauge EUS-FNB needles and 19-gauge conventional FNA needles.

## 2. Materials and Methods

### 2.1. Study Design

This was a retrospective study performed at a single tertiary care referral center where more than 100 EUS-FNBs are performed yearly (Toyama University Hospital). The study protocol was approved by the institutional review board of each participating institution (R2020130, Toyama University Hospital) and registered at the university hospital medical information network (UMIN) clinical trials registry (UMIN ID: UMIN000044058).

### 2.2. Patients

**Figure 1:**
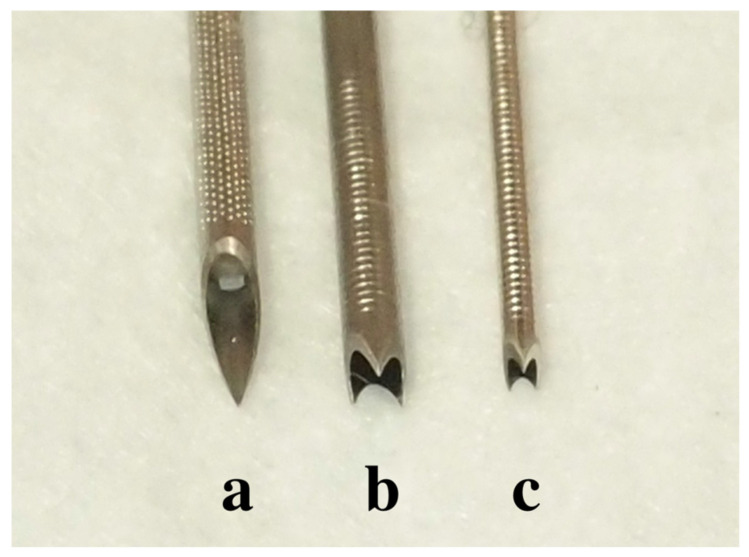
FNB and FNA needles (**a**) 19-gauge EZshot3^®^. (**b**) 19-gauge Acquire^®^. (**c**) 22-gauge Acquire^®^). FNB, fine-needle biopsy; FNA, fine-needle aspiration.

We performed EUS-FNA or FNB using three types of needles: 19-gauge and 22-gauge Franseen needles (Acquire, Boston Scientific, Natick, MA) and a 19-gauge conventional FNA needle (EZ Shot 3 Plus, Olympus, Tokyo, Japan) (Figure 1).

Among patients who underwent EUS-FNB between June 2018 and March 2020, 10 patients were randomly selected to evaluate the three needles. The choice of needle depended on the performance period. Initially, we used a 19-gauge conventional needle [11], followed by a 22-gauge Franseen needle. Later on, however, we used a 19-gauge needle for genetic analysis. It has been reported that the diagnostic accuracy of EUS-FNA for pancreatic cancer is lower than that for other pathologies [6]. In addition, the use of different puncture routes can affect the tissue acquisition. To minimize potential confounding and selection biases among the groups, we only included patients with suspected pancreatic cancer with a tumor size of ≥20 mm located in the body and tail of the pancreas.

### 2.3. Procedure

EUS was performed as an in-patient procedure using a curved linear echoendoscope (GF-UCT260; Olympus Corporation, Tokyo, Japan) connected to an ultrasound scanning system (EU-ME2; Olympus Corporation). The patients underwent EUS-FNA or FNB under conscious sedation with midazolam and their vital signs were monitored. Two experienced endosonographers (who perform the procedure for more than 50 cases per year) conducted the EUS-FNB in all cases. The procedure was performed as previously reported [12]. The lesion was punctured via the stomach or duodenum under guidance from real-time EUS imaging with color Doppler. After the stylet was removed, a 20 mL syringe was attached to the needle and 10 mL of negative pressure was applied. Several movements were made within the lesion and the suction was slowly released after the movements were completed. The needle was then withdrawn into the sheath and the entire system was withdrawn from the biopsy channel. The aspirated material was expelled onto glass slides by carefully reinserting the stylet into the needle. All patients were observed for ≥24 h after the procedure.

The specimen was then macroscopically evaluated and the whitish portions (macroscopically visible core) were collected and placed on a small piece of filter paper. The sample was then placed in formalin solution for histological examination and the remaining material was smeared on glass slides for cytological examination.

As our institution does not have an on-site pathologist or cytologist, punctures were repeated up to three times until a whitish material was macroscopically observed. If the third puncture was not successful, the lesion was diagnosed based only on the histological examination.

### 2.4. Histological Evaluation

All the collected specimens were sent for histological examination. After formalin fixation, the specimens were embedded in paraffin, sectioned, and subjected to hematoxylin-eosin staining and appropriate immunostaining according to the suspected diagnosis. All the histological diagnoses were made by two pathologists who specialized in the pancreatobiliary field at Toyama University Hospital. Final diagnoses were based on surgical pathology or a minimum six-month clinical follow-up.

**Figure 2:**
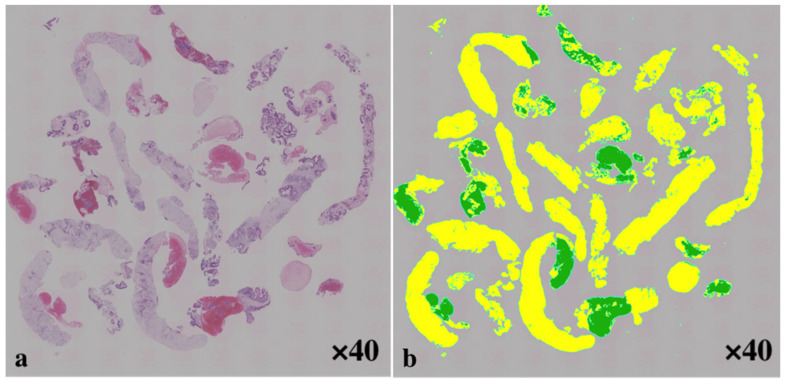
Histological evaluation (**a**) Hematoxylin and eosin staining (H and E, ×40) of a gross specimen obtained from the pancreatic cancer using a 22-gauge Franseen needle, viewed in a low power field. (**b**) Measuring the area of the specimen (green), excluding the blood clots (yellow), using imaging software (BZ-X800; Keyence Corporation, Osaka, Japan).

In this study, 12 of the total of 30 patients underwent surgical resection for pancreatic cancer, while the rest were diagnosed by disease progression or death due to the primary disease. The areas of the histological tissue and blood clots were measured separately using imaging software (BZ-X800; Keyence Corporation, Osaka, Japan) for each EUS-FNA and FNB pass (Figure 2). The data from the pathological samples with the largest tissue area were used.

### 2.5. Statistical Analyses

All the analyses were performed using JMP^®^ software (version 15; SAS Institute, Inc., Cary, NC, USA). The χ^2^ test and Fisher’s exact test were used to compare categorical parameters, and the Steel–Dwass test was used to compare continuous variables. Continuous parameters are presented as the median (IQR). *p* < 0.05 was considered statistically significant.

## 3. Results

Baseline characteristics of patients and solid pancreatic lesion details are listed in Table 1. All patients were diagnosed with pancreatic cancer with a tumor size of ≥20 mm located in the body and tail of the pancreas. There were no differences between each group in the mean age or gender of the patients or the median size of the lesions.

The EUS-FNA and FNB outcomes and pathological sample data are shown in Table 2. All procedures were performed through a transgastric puncture route. The number of puncture route needle passes using the 19-gauge conventional end-cut-type needle and the 22-gauge Franseen needle was two. Regarding the 19-gauge Franseen needle, the number of needle passes was only 1 in 2 of 10 cases. In all groups, the accuracy rate was 100%, and no adverse events were observed. The 19-gauge Franseen needle obtained significantly more histological tissue samples than the 19-gauge conventional (*p* = 0.010) and 22-gauge Franseen (*p* = 0.008) needles. Conversely, the difference between samples from the 19-gauge conventional needle and the 22-gauge Franseen needle was nonsignificant (*p* = 0.838).

Regarding the median area of blood clots, there were no significant differences among the groups (19-gauge Franseen needle vs. 19-gauge conventional needle, *p* = 0.162; 19-gauge Franseen needle vs. 22-gauge Franseen needle, *p* = 0.111; 19-gauge conventional needle vs. 22-gauge Franseen needle, *p* = 0.981). In addition, there were no significant differences among the groups in terms of the rate of the blood clots area/total area (19-gauge Franseen needle vs. 19-gauge conventional needle, *p* = 0.910; 19-gauge Franseen needle vs. 22-gauge Franseen needle, *p* = 0.910; 19-gauge conventional needle vs. 22-gauge Franseen needle, *p* = 1.000).

**Figure 3:**
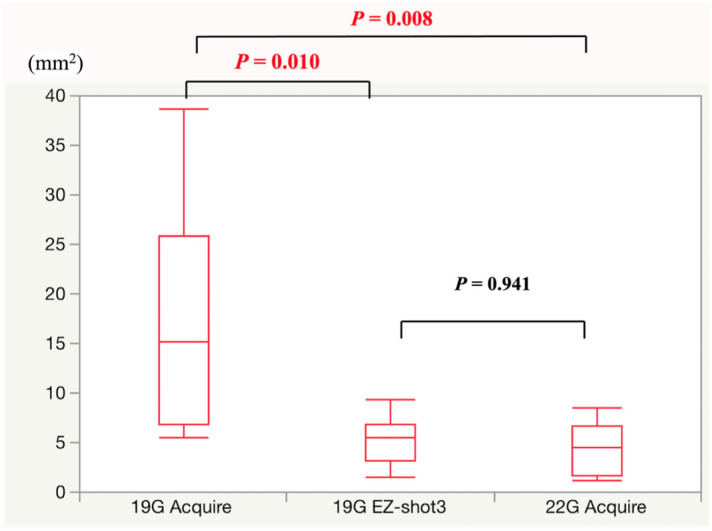
Area of the collected specimen measured by a Keyence digital microscope.

A scatter plot showing the amount of tissue obtained is shown in Figure 3. The amount of obtained tissue with the 19-gauge Franseen needle was approximately three times as large as that obtained with the 19-gauge conventional needle (median, 15.20 μm^2^ vs. 5.44 μm^2^, *p* = 0.010) and that obtained with 22-gauge Franseen needle (median, 15.20 μm^2^ vs. 4.49 μm^2^, *p* = 0.008).

## 4. Discussion

In the present study, we demonstrated that 19-gauge Franseen needles obtained significantly more histological tissue than 19-gauge conventional and 22-gauge Franseen needles among patients with pancreatic cancer. There were no differences among the groups regarding the median area of blood clots or the blood clots area/total area.

The pathological diagnosis of solid pancreatic masses is important for ensuring that suitable therapy is administered and the optimum prognosis is achieved [13,14]. Obtaining a large amount of tissue sample for pancreatic masses offers several advantages in addition to pathological diagnosis.

First, a larger amount of tissue provides pathologists with more detailed pathological information. It has been reported that the obtainment of only a small amount of a fragmented atypical epithelial cluster may make it difficult for a general pathologist to differentiate the cancer cells from gastric foveolar epithelial cells or normal pancreatic acinar cells that have atrophied due to inflammation [15,16]. In addition, Mukai et al. reported that a 22-gauge needle contributed to obtaining a pathological diagnosis, including the presence or absence of desmoplastic reactions. Moreover, a larger amount of tissue can provide the pathologist with information on venous, lymphatic, or nerve invasion [9]. If enough tissue is collected such that immunohistochemical assays, such as p53 or Ki-67, are not required, the time until diagnosis and the medical cost could be reduced.

Second, collecting a large amount of biopsy tissue may allow macroscopic assessment, such as macroscopic on-site evaluation (MOSE). Although a large amount of sample does not always guarantee a good macroscopic evaluation, it has the potential to lead to more accurate evaluations. The rapid on-site cytological evaluation (ROSE) of EUS-FNA samples performed by a cytopathologist influences the diagnostic performance of EUS-FNA, along with the location, size, and tissue firmness of the lesion, and the experience of the endoscopist [17,18,19]. If ROSE is not available, MOSE is a feasible method for achieving a high diagnostic accuracy and reducing the number of needle passes [11]. Our previous study revealed that the use of a Franseen needle provided sufficient tissue samples for histological interpretation in 96% of cases with one needle pass and in 100% of cases with three needle passes [20]. The Franseen needle enables MOSE even when using a 22-gauge needle, because of its ability to obtain a visible core. In this study, MOSE could be performed for all patients, resulting in a low number of passes (average, two passes) with a good diagnostic accuracy rate.

Third, recent molecular pathological studies have been performed for various types of cancer. Unfortunately, pancreatic cancer typically has low-cellularity tumors with abundant stromal components. The content of tumor cells in pancreatic cancer is reported to be approximately 5–20%, which makes the analysis of next-generation sequencing (NGS) relatively difficult [21]. Therefore, obtaining a sufficient amount of tissue for NGS analysis in addition to diagnosis is required. Elhanafi et al. compared EUS-FNA to FNB for the genomic analysis of pancreatic cancer. FNB resulted in a higher proportion of patients with sufficient samples compared to FNA (90.9% vs. 66.9%; *p* = 0.02). In multivariable modeling, only FNB (odds ratio, 4.95; 95% confidence interval, 1.11–22.05; *p* = 0.04) was associated with sufficient sampling for genomic testing [22].

Franseen needles have emerged as a preferred alternative to conventional needles, as they can easily obtain larger tissue samples. For example, Mukai et al. reported that a 22-gauge Franseen needle provides approximately five times the median area of tissue sample for histological evaluation compared to that of the same gauge conventional needle [9]. In this context, large samples maintain tissue architecture and can provide a more accurate pathological diagnosis compared to smaller samples. Furthermore, several studies have already confirmed the efficacy of Franseen needles [20,23,24].

**Figure 4:**
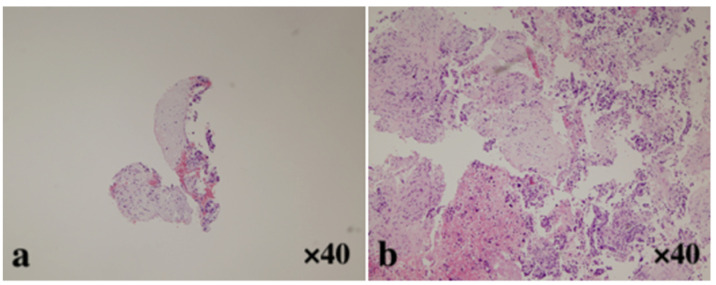
Comparison of histological sample volumes among various needles (**a**) A small amount of fragmented specimen obtained using 22-gauge FNA needles (H and E, ×40), which makes it difficult to diagnose adenocarcinoma. (**b**) A core tissue including the desmoplastic fibrosis with neoplastic cellular elements obtained using a 19-gauge FNB needle (H and E, ×40). This sample was obtained in sufficient quantity for genetic panel profiling testing for pancreatic cancer (OncoGuide^TM^ NCC Oncopanel system, Sysmex Corporation, Hyogo, Japan).

In the present study, there was no difference in histological tissue samples between the 22-gauge Franseen needle and the 19-gauge conventional needle (*p* = 0.838). This suggests that 22-gauge Franseen needles may have the same diagnostic ability as 19-gauge conventional needles. In addition, the amount of obtained tissue with a 19-gauge Franseen needle was approximately three times as large as that obtained with a 19-gauge conventional needle or with a 22-gauge Franseen needle. This suggests the ability of the 19-gauge Franseen needle to obtain larger tissue samples (Figure 4).

A concern with FNB needles that collect large amounts of tissue is the potential for more adverse events to occur. Bang et al. reported one bleeding adverse event (3.3%) consisting of arterial mucosal bleeding that required endoscopic hemostasis using two clips [10]. Mukai et al. reported that oozing from the puncture site appeared to increase with the Franseen needle, although no bleeding requiring hemostasis occurred [9]. In the present study, there were no differences among the groups regarding the median area of blood clots and the rate of the blood clots area/total area. However, the bleeding area tends to be relatively large for 19-gauge FNB needles, which suggests that needle puncture should be performed with great care to avoid intervening vessels.

The present study had a few limitations. First, the retrospective single-center study design and a small number of patients are associated with risk of bias. In this study, to minimize potential confounding and selection biases among the groups, we only included patients suspected of pancreatic cancer with a tumor size of ≥20 mm located in the body and tail of the pancreas. Under these conditions, the highest priority was to make the characteristics of all the subjects as equal as possible. As a result, each group had 10 cases. However, there is still a potential risk of selection bias. Second, the choice of the needle mainly depended on the performance period. Initially, we used a 19-gauge conventional needle [11], followed by a 22-gauge Franseen needle. Later on, we used a 19-gauge Franseen needle for genetic analysis. Third, in the present study various needles were compared only in the patients with pancreatic adenocarcinoma. It is unclear whether these results are applicable to other pancreatic masses, such as neuroendocrine tumors, autoimmune pancreatitis, or extra-pancreatic masses such as gastrointestinal stromal tumors. Third, this study excluded both small lesions (<20 mm) and transduodenal puncture routes. We previously reported that lesion size was an independent predictor of histopathological diagnosis accuracy with a 22-gauge Franseen needle using a multivariate analysis [25]. Regarding a transduodenal puncture route, there are several technical issues with the use of a large-caliber 19-gauge needle due to its stiffness. This may affect tissue collection due to poor maneuverability. In the present study, these issues were not considered. Finally, genomic assessment was not performed in this study. It is unclear whether the Franseen needle has advantages for collecting tissue for genomic profiling. We are currently conducting another prospective cohort study on the diagnostic quality of EUS-FNB for an onco-panel for the genomic profiling of pancreatic cancer.

In conclusion, the 19-gauge Franseen needle can obtain samples approximately three times larger than those of other needles, which can be useful for obtaining a more precise pathological diagnosis and information, including genomic profiling.

## Figures and Tables

**Table 1:** Baseline characteristics and pancreatic solid lesions details.

	19-Gauge Franseen (*n* = 10)	19-Gauge Conventional End-Cut Type (*n* = 10)	22-Gauge Franseen (*n* = 10)
Mean age (range), years	70.5 (48–84)	72 (67–85)	71.5 (50–82)
Gender, no.			
Male/female	6/4	3/7	4/6
Mass location, no.			
Head/uncinate	0	0	0
Body/tail	10	10	10
Size of masses on EUS, mm			
Median (range)	36.2 (24.1–53.1)	36.5 (20.5–38.8)	30.7 (21.8–39.7)
Final diagnosis			
Pancreatic adenocarcinoma	10	10	10

EUS, endoscopic ultrasonography.

**Table 2:** Outcomes of EUS-FNA and FNB and data of pathological samples.

	19-Gauge Franseen (*n* = 10)	19-Gauge Conventional End-Cut Type (*n* = 10)	22-Gauge Franseen (*n* = 10)
Procedure			
Expert/Trainee	0/10	0/10	0/10
Puncture route			
Transgastric/Transduodenal	10/0	10/0	10/0
Number of passes, mean (range)	2 (1–2)	2 (2)	2 (2)
Diagnostic yield, *n* (%)	10 (100)	10 (100)	10 (100)
Adverse events, *n* (%)	0 (0)	0 (0)	0 (0)
Area of the pathological tissue, median, mm^2^ (IQR)	15.20 (6.89–25.75) ^(a) (b)^	5.44 (3.19–25.75) ^(a)^	4.49 (1.69–6.63) ^(b)^
Area of blood clots, median, mm^2^ (IQR)	1.81 (1.14–3.14)	0.85 (0.35–1.59)	0.53 (0.24–1.76)
Rate of the blood clots area/total area, % (IQR)	17.0 (3.8–31.5)	17.5 (4.0–25.8)	8.0 (3.8–47.5)

FNA, fine-needle aspiration; FNB, fine-needle biopsy needle; IQR, interquartile range. ^(a)^ *p* = 0.010, ^(b)^ *p* = 0.008.

## Data Availability

No new data were created or analyzed in this study. Data sharing is not applicable to this article.
